# Visual nutrition analysis: leveraging segmentation and regression for food nutrient estimation

**DOI:** 10.3389/fnut.2024.1469878

**Published:** 2024-12-17

**Authors:** Yaping Zhao, Ping Zhu, Yizhang Jiang, Kaijian Xia

**Affiliations:** ^1^School of Artificial Intelligence and Computer Science, Jiangnan University, Wuxi, Jiangsu, China; ^2^Changshu Key Laboratory of Medical Artificial Intelligence and Big Data, Suzhou, Jiangsu, China; ^3^Department of Scientific Research, The Changshu Affiliated Hospital of Soochow University, Suzhou, Jiangsu, China

**Keywords:** nutrition estimation, Nutrition5k, deep learning, image segmentation, regression

## Abstract

**Introduction:**

Nutrition is closely related to body health. A reasonable diet structure not only meets the body’s needs for various nutrients but also effectively prevents many chronic diseases. However, due to the general lack of systematic nutritional knowledge, people often find it difficult to accurately assess the nutritional content of food. In this context, image-based nutritional evaluation technology can provide significant assistance. Therefore, we are dedicated to directly predicting the nutritional content of dishes through images. Currently, most related research focuses on estimating the volume or area of food through image segmentation tasks and then calculating its nutritional content based on the food category. However, this method often lacks real nutritional content labels as a reference, making it difficult to ensure the accuracy of the predictions.

**Methods:**

To address this issue, we combined segmentation and regression tasks and used the Nutrition5k dataset, which contains detailed nutritional content labels but no segmentation labels, for manual segmentation annotation. Based on these annotated data, we developed a nutritional content prediction model that performs segmentation first and regression afterward. Specifically, we first applied the UNet model to segment the food, then used a backbone network to extract features, and enhanced the feature expression capability through the Squeeze-and-Excitation structure. Finally, the extracted features were processed through several fully connected layers to obtain predictions for the weight, calories, fat, carbohydrates, and protein content.

**Results and discussion:**

Our model achieved an outstanding average percentage mean absolute error (PMAE) of 17.06% for these components. All manually annotated segmentation labels can be found at https://doi.org/10.6084/m9.figshare.26252048.v1.

## 1 Introduction

Nutritional content in the diet is closely related to health. Our bodies need various nutrients to maintain normal physiological functions, with each nutrient having specific roles. A deficiency or excess intake can negatively impact health (1). A reasonable diet structure not only meets the body’s nutritional needs but also prevents many chronic diseases. For example, a high-fiber diet can reduce the risk of heart disease and diabetes (2), while foods rich in antioxidants can help prevent certain types of cancer.

In modern society, many people have irregular eating habits due to a fast-paced lifestyle. High-fat, high-sugar, and high-salt foods dominate diets, often driven by convenience and availability. These unhealthy dietary patterns increase the risk of obesity, hypertension, and cardiovascular diseases, posing significant public health challenges. Consequently, understanding and selecting healthy foods, as well as maintaining a balanced nutritional intake, has become increasingly important. However, understanding the specific nutritional content of food is a complex and time-consuming task for the average person. Determining nutritional content typically requires knowledge of food composition, the use of food scales, and extensive manual calculations, which can be a deterrent for many. Simplifying this process by directly predicting nutritional content from food images could provide an efficient and accessible solution, especially for individuals seeking to improve their dietary habits.

Currently, common dietary calorie and nutrition calculation applications mainly provide users with channels for nutrition inquiry and record-keeping. Users must provide the exact type and weight of each food, which the application uses to calculate specific nutritional content. This process requires using a scale to weigh food for more accurate results. However, this method is time-consuming and laborious, often leading users to estimate food portions visually, resulting in inaccurate quantity calculations (3). Additionally, these applications offer a limited variety of dishes for inquiry, and many times our food cannot accurately match the existing menu, greatly reducing the accuracy of nutritional content prediction.

Image-based nutritional content prediction has gained significant attention as an innovative approach to address these challenges. Using mobile phone cameras to analyze food images and estimate nutritional content can streamline the process, eliminating the need for extensive manual input. This technology not only enhances convenience but also provides insights into foods not included in traditional databases, offering personalized dietary recommendations based on actual meals. By integrating visual recognition with nutritional analysis, such methods could revolutionize dietary monitoring and promote healthier eating habits.

In recent years, research in computer vision has made substantial progress in food recognition, classification, and segmentation. For example, Jiang et al. (4) proposed a three-step algorithm using deep convolutional neural networks (CNNs) to recognize multi-food images, generating proposal regions through a region proposal network (RPN) and then classifying and locating these regions. In nutritional content prediction, they preset the weight of each food to calculate nutritional information. Similarly, Situju et al. (5) used food image classification methods to estimate food calorie and salt content, employing a two-stage transfer learning method to enhance food component estimation. However, these studies focus on food classification, calculating nutritional information by classifying food into known menu items and assuming that foods classified as the same dish have roughly the same nutritional content.

Many studies focus on calculating food area or volume. For example, Meyers et al. (6) segmented food images to distinguish different food regions and converted 2D images into 3D volume representations to calculate food calories. Agarwal et al. (7) proposed a hybrid architecture primarily utilizing the Mask RCNN and YOLO V5 frameworks. Through the processes of segmentation, classification, and calculating the volume and calories of food items, it predicts the calorie content of food in a bowl. Yang et al. (8) used an AI system mimicking the thinking of human nutritionists, learning the volume of common objects (e.g., teaspoons, golf balls, cups) to “mentally” measure food volume. However, this method is limited to situations where only one type of food is on a plate. Raju et al. (9) enhanced hardware by proposing a new passive, independent, multi-spectral, motion-activated, structured light-supplemented stereo camera (FOODCAM) for food intake monitoring, capable of capturing and reconstructing 3D images of complex-shaped foods. However, this equipment is too complex for practical use.

Despite these advancements, many models still rely on classifying food into predefined dish types to estimate nutritional content, which introduces significant uncertainty due to the varying densities and compositions of foods. Moreover, the lack of real nutritional content labels in most datasets further undermines accuracy and generalizability.

Therefore, we aim to combine image segmentation and nutritional content prediction. Currently, most existing datasets focus on increasing the number of food categories or improving food segmentation accuracy. For example, Recipe1M (10) is a dataset generated from images scraped from recipe websites, containing ingredients used in dishes. However, these annotations do not fully correspond to the images, as the quantities of ingredients shown in the images are not explicitly marked. Thus, directly using this dataset for nutritional content prediction may not yield accurate results.

UEC-FoodPix (11) and UEC-FoodPix Complete (12) are large-scale food image segmentation datasets derived from UEC-Food100 (13). UEC-FoodPix adds semi-automatic segmentation templates to existing food images, and UEC-FoodPix Complete manually refines them. Since most images in this dataset contain rice, the authors estimated the food area through rice grains to calculate food calories.

Methods predicting nutritional content by estimating food area or volume cannot measure accuracy without real nutrition labels. Nutrition5k (14) is a dataset collected in cafeterias, using professional instruments to weigh, scan, and record each dish, containing 5,000 different dish video streams and depth images. Each dish has detailed ingredient and content labels, with calorie, fat, protein, and carbohydrate content calculated using the USDA Food and Nutrient Database (15), providing highly accurate nutritional information.

Nutrition5k is one of the few datasets that provides complete nutritional information and content for the corresponding food in images. Research on the relationship between food images and nutritional content based on Nutrition5k mainly falls into three directions.

One approach involves estimating food content based on volume or area, such as the method proposed by Shao et al. (16)., who introduced an end-to-end food portion estimation framework based on monocular image 3D shape reconstruction. This method estimates the energy value of food from monocular images using deep learning. This approach primarily focuses on food images, but does not fully utilize the nutritional information provided by the dataset. Another approach is to sequentially perform food classification followed by nutrient content prediction. For example, Wang et al. (17) integrated EfficientNet, Swin Transformer, and Feature Pyramid Network (FPN) to develop an efficient, lossless, and accurate method for food nutrient recognition and quantification. A third approach is to predict nutrition by fusing raw images and depth images after a series of processing steps. For example, Shao et al. (18) uses raw RGB images and depth images captured by special equipment, integrating multimodal feature fusion (MMFF) and multi-scale fusion for vision-based nutrition assessment. Han et al. (19) proposes an end-to-end nutrition estimation method based on monocular images, DPF-Nutrition. By introducing a depth prediction module to generate depth maps, this method improves the accuracy of food content estimation. While this approach effectively utilizes depth information and eliminates the need for volume reconstruction, the process of simultaneously handling both color and depth images significantly increases memory overhead.

We aim to make effective use of the Nutrition5k dataset while minimizing or avoiding the shortcomings of the above methods. Therefore, we selected 3,224 top-view food images from Nutrition5k, manually adding segmentation labels to build a model that segments first and predicts later, along with corresponding detailed nutritional content labels. This model first segments the food portion from the original image and then predicts the content of weight, calories, fat, carbohydrates, and protein. Our method achieved an average percentage mean absolute error (PMAE) of 17.06% for these five components.

The innovations of this paper are as follows:

• Dataset Selection and Annotation: We selected 3,224 food images from the Nutrition5k dataset and manually segmented them. These images and their detailed ingredient labels can be used for nutritional content prediction tasks.
• Segmentation-First Prediction Framework: We constructed a segmentation-first prediction framework. First, the UNet network segments the original image, and the resulting pure food image is feature-extracted through a backbone network, further enhancing feature expression through the SE module. The enhanced features are input into a series of fully connected layers to output the predicted values of food weight, calories, fat, carbohydrates, and protein. This method accurately segments the food area and efficiently extracts and utilizes image features, improving the accuracy of nutritional content prediction.
• Reducing Environmental Impact: By segmenting the food in the image before predicting nutritional content, the method effectively reduces the impact of surrounding environments, focusing the model on the food itself.
• Using Real Nutritional Content Labels: We use real nutritional content labels as training data, and the results on the test set can be compared with real data. Compared to previous methods focusing on food area or volume calculation, our approach is more realistic and practical.

The structure of this paper is as follows: Chapter 1 introduces the research background, objectives, and innovations. Chapter 2 describes the dataset and methods used in the study, including data sources and selection, ingredient label annotation methods, and the overall network architecture. Chapter 3 presents experimental results, starting with food segmentation effects and then detailing nutritional content prediction results, including specific details of the experimental setup, comparative analysis of experimental results, and ablation experiments to verify the effectiveness of model components. Chapter 4 discusses the significance and limitations of the research results and provides some dietary plan guidelines. Chapter 5 summarizes and concludes the content of this paper.

## 2 Materials and method

### 2.1 Dataset

#### 2.1.1 Source Dataset

We used data from the Nutrition5k dataset collected by Thames et al. (14). This dataset, collected from campus cafeterias using professional instruments, contains 5,000 real-world food dishes with video streams, depth images, ingredient weights, and high-precision nutritional content annotations. The dataset aims to provide diverse, realistic, and challenging data for improving visual nutrition estimation by capturing authentic cafeteria food photos.

During data collection, dishes were added to plates or bowls item by item. Each addition was recorded as a new entry, with each recorded item having a detailed ingredient list. The detailed ingredient information for each dish was obtained based on the recorded weights. Subsequently, the USDA Food and Nutrient Database (15) was used to calculate the calorie, fat, protein, and carbohydrate content of each dish.

Ultimately, the dataset comprises over 5,000 dishes made up of more than 250 different ingredients, ranging from 1 to 35 ingredients per dish, with an average of 5.7 ingredients per dish. These dishes span from a few calories to over 1,000 calories each. Each dish has detailed ingredient and content labels. Among these, 3,500 dishes also include top-view images and top-down RGB-D images taken using an Intel RealSense camera.

We used data primarily from 3,490 top-view images and their ingredient labels from Nutrition5k. Compared to existing datasets, Nutrition5k provides more accurate ingredient content and nutritional information annotations. These annotations were obtained through step-by-step weighing, scanning, and recording of each ingredient in an actual cafeteria, using custom sensor arrays to scan and weigh each dish, ensuring high data accuracy.

#### 2.1.2 Data screening, annotation and analysis

**FIGURE 1:**
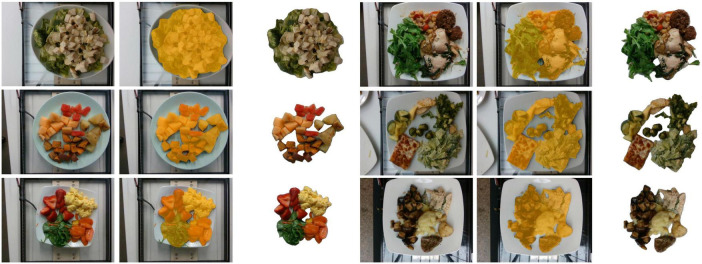
Original food images and their segmentation labels. Columns 1 and 4 show the original images, columns 2 and 5 show images with segmentation labels overlaid on the original images, and columns 3 and 6 show the segmented food images (foreground images).

The dataset contains 3,490 top-view RGB images, which we manually screened, ultimately selecting 3,224 images for all subsequent experiments. The original images, images with segmentation labels, and segmented images are shown in Figure 1.

**FIGURE 2:**
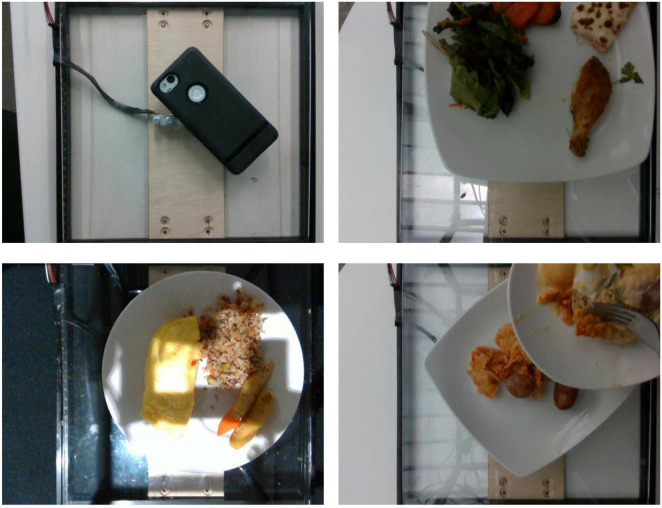
Some excluded data. The top left image is non-food, the top right image is misaligned, the bottom left image has overly uneven lighting, and the bottom right image shows overlapping dishes.

We first annotated all original images with segmentation labels. We used EISeg (20), an interactive segmentation annotation software developed based on PaddlePaddle. During the segmentation annotation process, we identified the following issues in some images: overly uneven lighting, misaligned shots causing parts of dishes to be out of frame, multiple occurrences of the same dish, and unrelated non-dish images. As shown in Figure 2, we excluded 250 problematic images in this stage.

**FIGURE 3:**
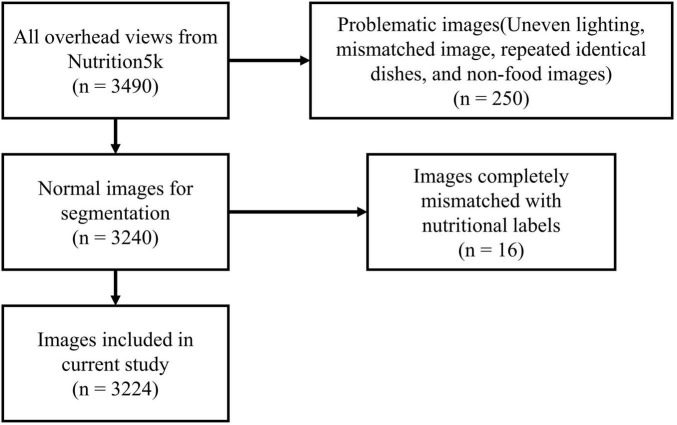
Data screening process.

After completing image screening and segmentation, we further checked the ingredient labels and found some data where images and ingredient labels did not match. We compared images and ingredient labels one by one to see if the foods appearing in the dishes matched the recorded ingredients and if there were any noticeable discrepancies in quality, ultimately excluding 16 entries. The complete data screening process is shown in Figure 3.

Finally, we used the EISeg tool to segment the food portions in the remaining 3,224 images, saving the segmentation results as JSON files, foreground images, binary images, and pseudo-color images. All segmentation labels have been publicly uploaded to https://doi.org/10.6084/m9.figshare.26252048.v1 (Note: the original dataset authors have open-sourced the data; please refer to the original text (14). We only uploaded the segmentation label files). Additionally, during the comparison of food images and nutritional content labels, we recorded detailed issues for each discarded entry, uploading these records along with the label files.

**FIGURE 4:**
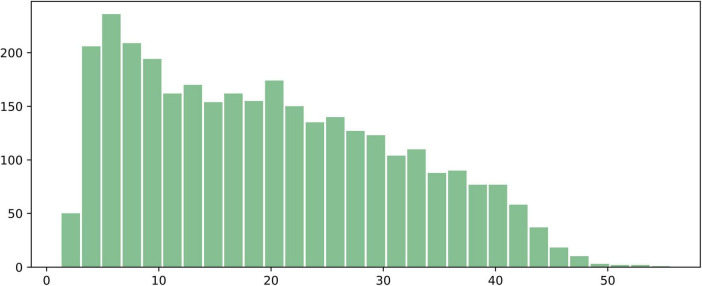
Histogram of the proportion of food regions in the 3,224 annotated images. The *x*-axis represents the ratio of the number of pixels occupied by the food to the total number of pixels in the image (%), and the *y*-axis represents the number of images corresponding to each ratio.

We analyzed 3,224 annotated images with food region segmentation labels and plotted a distribution histogram of the food area ratio in the images, as shown in Figure 4. The *x*-axis represents the ratio of the number of pixels occupied by the food to the total number of pixels in the image (%), and the *y*-axis represents the number of images corresponding to each ratio. As can be seen, the majority of food regions occupy between 5 and 30% of the image area. After 30%, the proportion of images with larger food areas gradually decreases, and very few samples have food regions occupying more than 50% of the image. A small number of food regions are smaller than 5% or larger than 50%, but overall, the distribution of food region areas is relatively even, with extreme cases being rare.

#### 2.1.3 Ingredient labels

**FIGURE 5:**
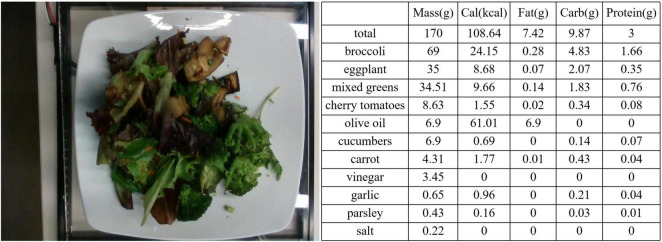
Label example. On the left is the image of dish_1559678127, and on the right are its corresponding ingredient labels.

All data used in this experiment can be represented as: $X={\left\{I_{i},{\text{Y}}_{i}\right\}}_{i=1}^{N}$, where *I*_*i*_ represents the image of the _*i*_-th data point, and *Y*_*i*_ represents the ingredient label of the _*i*_-th data point, with a total of _*N*_ such data points. The ingredient label$Y_{i}=\left(y_{i}^{m\,a\,s\,s},y_{i}^{c\,a\,l\,o\,r\,i\,e},y_{i}^{c\,a\,r\,b},y_{i}^{f\,a\,t},y_{i}^{p\,r\,o\,t\,e\,i\,n}\right)$ is a five-element vector, where these elements, respectively, represent the weight, calorie content, carbohydrate content, fat content, and protein content of sample *i*. Among these, $y_{i}^{m\,a\,s\,s}$ (in grams) is recorded during data collection, while $y_{i}^{c\,a\,l\,o\,r\,i\,e},y_{i}^{c\,a\,r\,b},y_{i}^{f\,a\,t},y_{i}^{p\,r\,o\,t\,e\,i\,n}$ are calculated based on the food type and $y_{i}^{w\,e\,i\,g\,h\,t}$ . In Figure 5, we show an example of a food image and its corresponding ingredient labels.

### 2.2 Method

**FIGURE 6:**
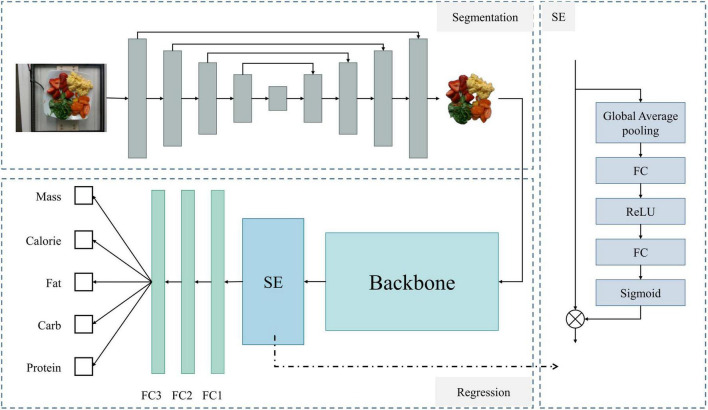
Overall architecture of the model. The upper left shows the network for food segmentation, the lower left shows the regression network for predicting food nutritional content, and the right side details the SE module’s structure.

To combine food segmentation and nutrient content prediction tasks, we constructed a network framework that first segments the image and then performs regression. This framework integrates segmentation and regression models to predict the weight, calories, fat, carbohydrates, and protein content. The overall structure of the network is shown in Figure 6.

First, for the segmentation network, we used the U-Net (21) architecture. U-Net consists of an encoder and a decoder. The encoder progressively extracts features from the image, while the decoder progressively restores the spatial resolution of the image through upsampling. Skip connections between the encoder and decoder pass high-resolution feature maps to retain more detailed information. Next is the regression model for predicting nutritional content. This part first uses a backbone network [ultimately ResNet-101 (22)] to extract feature information. The Squeeze-and-Excitation (23) structure captures critical information from the input features, enhancing the network’s expressive power. Finally, three fully connected layers output the final predictions for calories, weight, carbohydrates, fat, and protein.

#### 2.2.1 Segmentation network

The first step of the model is to segment the input food image, separating the food portion from the background. For this, we used the U-Net segmentation network. U-Net has a symmetrical encoder-decoder structure that effectively captures multi-scale features of the image.

The U-Net network structure is shown in the upper left dashed box of Figure 6. For an input food image, it first undergoes a series of convolutional and pooling layers for downsampling, progressively extracting high-level features. Then, a series of upsampling and convolution operations gradually restore the spatial resolution. At each upsampling step, U-Net concatenates the corresponding downsampled feature maps with the current upsampled feature maps through skip connections. After four stages of encoding and decoding, the feature maps are adjusted to the same size as the original image. Finally, a convolution layer maps the feature maps to the desired number of output channels (i.e., the number of segmentation categories).

Using U-Net for food segmentation has the following advantages: First, the skip connection mechanism of U-Net can retain high-resolution features from the encoding stage during decoding, ensuring that the segmentation results have fine boundary information, which is crucial for complex shapes and details in food segmentation. Second, U-Net has a simple structure and fewer parameters, achieving good segmentation results even with limited data. Although we ultimately used 3,224 images for training and testing, the model already showed excellent results. Additionally, the skip connections in U-Net facilitate gradient backpropagation, allowing the network to converge faster and training to be more efficient.

#### 2.2.2 Regression network

The segmented food images are used in the regression network to predict nutritional content. The model for this part is shown in the lower left of Figure 6 (Regression section). First, we use a backbone network to extract feature information, which can be any feature extraction network. After several experiments, we selected ResNet-101 as the backbone network. The features extracted by the backbone are enhanced using the Squeeze-and-Excitation (SE) structure and finally passed through three fully connected layers to obtain individual outputs for each nutritional component.

The SE module selectively emphasizes useful features and suppresses less useful ones through two stages: Squeeze and Excitation. The model diagram is shown on the right side of Figure 6. In the Squeeze stage, global average pooling compresses the spatial information of each channel into a single value, generating channel-level descriptors. This step can be expressed as:


(1) $$z_{c}=\frac{1}{H\times W}\,\sum_{i=1}^{H}\sum_{j=1}^{W}X_{i\,j\,c}$$

Where *X*_*ijc*_ represents the value at position (*i*,*j*) in the *c*-th channel of the input feature map. *H* and *W* denote the height and width of the feature map, and *z*_*c*_ is the descriptor for the *c*-th channel.

Next is the Excitation stage, which includes two fully connected layers and two activation functions. The first fully connected layer compresses the number of channels, and the second fully connected layer restores the number of channels, using the learned weights to excite the original feature map:


(2) $$s=\sigma_{2}\,\left(W_{2}\cdot \sigma_{1}\,\left(W_{1}\cdot z\right)\right)$$

where *z* represents the vector of descriptors for all channels, *W*_1_ and *W*_2_ are the weight matrices of the two fully connected layers, σ_1_ is the ReLU activation function, σ_2_ is the Sigmoid activation function, and *s* represents the channel weights obtained through the excitation process.

Finally, each channel in the original feature map is multiplied by the corresponding weight to complete feature recalibration (denoted by ⊗ in the model diagram):


(3) $${\tilde{X}}_{c}=s_{c}\cdot X_{c}$$

where ${\tilde{X}}_{c}$ is the recalibrated feature of the *c*-th channel, and *s*_*c*_ is the weight of the *c*-th channel.

The recalibrated features emphasize the more important parts of the feature channels, improving the accuracy of subsequent nutritional content predictions.

## 3 Results

### 3.1 Food segmentation

First, we trained the model on segmentation using the annotated data. During this process, besides using the UNet model, we also compared it with the classic semantic segmentation models DeepLabv3 (24) and FCN (25). We randomly split the 3,224 original data instances into training and test sets in an 8:2 ratio. The three models were trained for 50 epochs on the training set and then evaluated on the test set to obtain results on four evaluation metrics: Dice, Jaccard, Precision, and Recall. The following are the formulas for these evaluation metrics:


(4) $$\text{Dice}=\frac{2\times \text{TP}}{2\times \text{TP}+\text{FP}+\text{FN}}$$

(5) $$\text{Jaccard}=\frac{\text{TP}}{\text{TP}+\text{FP}+\text{FN}}$$

(6) $$\text{Precision}=\frac{\text{TP}}{\text{TP}+\text{FP}}$$

(7) $$\text{Recall}=\frac{\text{TP}}{\text{TP}+\text{FN}}$$

where TP is the number of pixels correctly predicted as food regions, FP is the number of pixels incorrectly predicted as food regions, FN is the number of pixels incorrectly predicted as non-food regions, and TN is the number of pixels correctly predicted as non-food regions. The final average test results on the test set are shown in Table 1.

**TABLE 1:** Results of all segmentation models on Dice, Jaccard, Precision, and Recall metrics.

Methods	Dice	Jaccard	Precision	Recall
DeepLabV3	92.40	86.19	88.89	96.55
FCN	95.52	91.67	95.20	96.09
UNet	97.69	95.52	97.40	98.02

UNet achieved the best results across all four evaluation metrics. As shown in Table 1, the UNet model performed the best on all evaluation metrics, with a Dice coefficient of 97.69%, an improvement of 2.17% over FCN and 5.29% over DeepLabV3. For the Jaccard index, UNet reached 95.52%, 3.85% higher than FCN and 9.33% higher than DeepLabV3. Additionally, UNet’s Precision was 97.40%, 2.20% higher than FCN’s 95.20%, and 8.51% higher than DeepLabV3’s 88.89%. In terms of Recall, UNet also excelled, achieving 98.02%, 1.93% higher than FCN’s 96.09%, and 1.47% higher than DeepLabV3’s 96.55%.

These results indicate that UNet not only leads in overall performance but also significantly outperforms FCN and DeepLabV3 on each specific metric. Particularly in the Dice coefficient and Jaccard index, which are key segmentation performance metrics, UNet shows higher segmentation accuracy and better region overlap. Furthermore, UNet demonstrates better balance in Precision and Recall, indicating its significant advantage in accurately and comprehensively identifying food regions. Overall, the UNet model performs the best in food segmentation tasks, providing higher quality segmentation results.

**FIGURE 7:**
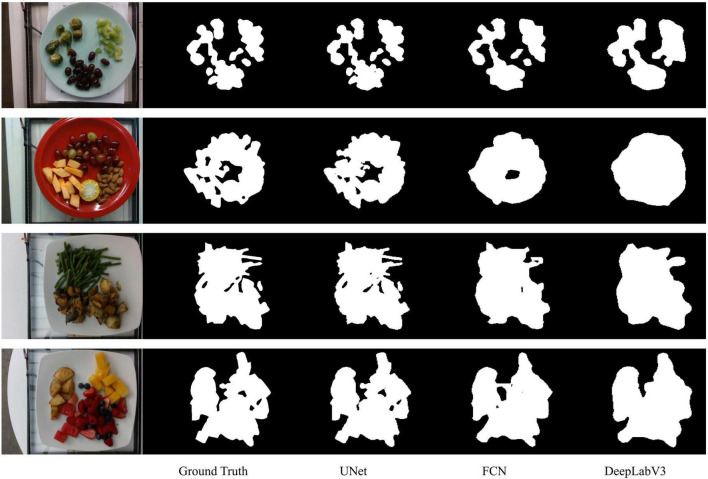
Comparison of visual results of food segmentation tasks using UNet, FCN, and DeepLabV3 models. The first column is the original image, the second column is the ground truth, the third column is the segmentation result of UNet, the fourth column is the segmentation result of FCN, and the fifth column is the segmentation result of DeepLabV3.

As seen in Figure 7, UNet captures more details when segmenting food, especially excelling at food edges. For foods with complex shapes and small parts, UNet better preserves their shape features. For example, in the upper part of the green beans in the third row of images in Figure 7, UNet can precisely segment the prominent slender parts. Additionally, UNet can also handle small food items outside the main food area. Even in the second row of images in Figure 7, where the food color is very close to the container color, UNet can exclude background interference and accurately segment the food region. Overall, UNet’s segmentation results are very close to the ground truth.

The FCN model can segment the main food regions well and accurately grasp the overall shape. Compared to UNet, the FCN model is slightly lacking in detail, with some edge parts and smaller food regions possibly missed or inaccurately segmented. Additionally, the FCN’s performance is slightly insufficient when dealing with foods whose color is very close to the background.

DeepLabV3 lags behind the other two models overall because it overly focuses on global information and overlooks some small local features, leading to less precise detail segmentation than UNet.

### 3.2 Nutrient prediction

#### 3.2.1 Experimental setup

##### 3.2.1.1 Implementation details

Building on the previous segmentation task, we conducted nutrient prediction experiments using pure food images obtained from UNet segmentation. For the images used in the regression task, the pixel values in regions outside of the food area were set to zero. All nutritional information was stored in a text file in the format [image_name, calories, mass, fat, carb, protein], with the nutrition information corresponding to the input images by image name.

All models were implemented using Python 3.7 and PyTorch 1.13, and we used the Adam optimizer with an initial learning rate. During training, the learning rate was adjusted using an exponential decay rule with a decay rate of 0.99. The default initial value for the random seed was 42. The experiments were conducted on a single NVIDIA GeForce RTX 4060 GPU. For all comparison models, we used official pre-trained weights (if available) for initialization and further training.

We randomly split the 3,224 data samples into training and testing sets at a ratio close to 8:2, with 2,576 samples assigned to the training set and 648 samples to the testing set (The split is not strictly 8:2 to ensure that both the training and testing sets have sample counts that are multiples of 8, which facilitates subsequent batch size division). Before inputting each training image into the regression network, random horizontal flipping and random cropping were applied as preprocessing steps to enhance image diversity and improve the model’s generalization ability.

The batch size for all experiments was set to 8, and each model was trained for 300 epochs with 322 iterations per epoch on the 2,576 training samples. After each epoch, the model was tested on the 648 test samples. The average training time for each model was about 6 h. Finally, we compared and analyzed the top-1 results for each model.

##### 3.2.1.2 Loss functions

During training, loss functions are used to measure the discrepancy between predicted and actual values, allowing for the adjustment of model parameters during backpropagation to minimize the loss. Since this experiment aims to predict five metrics (mass, calories, fat, carbohydrate, protein), we divided them into five subtasks. Let *M* = {*weight*,*cal*,*fat*,*carb*,*protein*} . For each subtask mmm (*m* ∈ *M*), the loss calculation formula is as follows:


(8) $$l_{m}=\frac{\sum_{i=1}^{N}\left|{\hat{y}}_{i}^{\text{m}}-y_{i}^{\text{m}}\right|}{\sum_{i=1}^{N}y_{i}^{\text{m}}}$$

where${\hat{y}}_{i}^{\text{m}}$ represents the predicted value for the *i*-th sample in subtask *m*, $y_{i}^{\text{m}}$ represents the actual value for the *i*-th sample in subtask *m*, and *N* represents the total number of samples. Compared to the more commonly used mean absolute error (MAE), this loss function calculates the sum of the absolute errors between predicted and actual values and normalizes it by the sum of the actual values.

This approach addresses the issue of the significantly higher magnitudes of mass and calorie values compared to fat, protein, and carbohydrate values. In most samples, the values of mass and calories are more than ten times those of fat, protein, and carbohydrates. Directly summing the losses of all subtasks would make it difficult to optimize the subtasks with lower magnitudes. By normalizing the absolute error, we avoid the impact of different magnitudes of metrics, allowing the losses of each subtask to be compared on the same scale. This processing method helps maintain balance among different metrics during training, improving the overall performance and prediction accuracy of the model.

The final total loss is:


(9) $$L=l_{\text{weight}}+l_{\text{cal}}+l_{\text{carb}}+l_{\text{fat}}+l_{\text{protein}}$$

where *l*_weight_,*l*_cal_,*l*_carb_,*l*_fat_,*l*_protein_ represent the losses for mass, calories, carbohydrates, fat, and protein, respectively.

To better measure the differences in nutrient prediction performance across various methods, we use PMAE as the evaluation metric. PMAE normalizes the MAE by dividing it by the mean of the actual values. This normalization effectively mitigates the impact of different magnitudes of the metrics, ensuring better comparability and consistency in error measurement across different metrics. A lower PMAE value indicates a smaller discrepancy between predicted and actual values, signifying a more accurate prediction by the model.

The formula for PMAE is as follows:


(10) $$\text{MAE}=\frac{1}{N}\,\sum_{i=1}^{N}\left|{\hat{y}}_{i}-y_{i}\right|$$

(11) $$\text{PMAE}=\frac{\text{MAE}}{\frac{1}{N}\,\sum_{i=1}^{N}y_{i}}$$

where ${\hat{y}}_{i}$ represents the predicted value for the *i*-th sample, *y*_*i*_ represents the actual value, and *N* denotes the total number of samples.

#### 3.2.2 Experimental results

When selecting the backbone, we experimented with several classical models, including various deep convolutional neural networks: the classic deep residual networks ResNet50 and ResNet101 (22), GoogLeNet (26), which uses Inception modules to enhance computational efficiency and accuracy, InceptionV3 (27), which further optimizes the Inception modules of GoogLeNet, DenseNet (28), which uses dense connections to alleviate the vanishing gradient problem and improve feature reuse and model efficiency, MobileNetv3 (29), a lightweight network optimized for mobile devices with efficient convolutional layers and attention mechanisms, VGG (30), a simple yet deep network structure widely used in image tasks, and ShuffleNetV2 (31), a lightweight network achieving efficient computation through channel shuffling and efficient convolution operations. Additionally, we also tried the Vision Transformer (ViT) (32) for processing image data using the Transformer architecture, including versions with patch sizes of 32 and 16.

To ensure fairness, all models were tested under the same experimental setup, with only the backbone network being replaced. If official pre-trained models were available, we used pre-trained weights for initialization before training on our dataset. The final experimental results are shown in Table 2.

**TABLE 2:** Comparison of PMAE results for calorie, mass, fat, carb, and protein between our method and all other methods, with the best values in bold.

Methods	Calorie PMAE (%)	Mass PMAE (%)	Fat PMAE (%)	Carb PMAE (%)	Protein PMAE (%)	Mean PMAE (%)
GoogLeNet	27.87	24.27	44.65	47.53	40.28	36.92
GoogLeNetV3	20.59	16.41	32	28.25	28.15	25.08
DenseNet121	19.67	14.53	30.33	24.77	26.4	23.14
ViTb32	17.78	14.83	27.8	23.9	24.2	21.7
MobileNetv3	18.98	13.91	28.85	23.13	23.24	21.62
ShuffleNetV2	18.11	13.94	28.29	23	23.54	21.37
ViTb16	16.83	13.32	24.87	21.2	21.37	19.52
VGG	16.21	12.02	24.66	19.29	20.44	18.52
Resnet50	16.48	11.87	24.18	19.9	19.24	18.33
Ours	15.68	**11.75**	**21.41**	**18.79**	**17.68**	**17.06**

The bold values represent the best value across all methods.

Our model, using ResNet101 as the backbone network, achieved PMAE values of 15.68% for calories, 11.75% for mass, 21.41% for fat, 18.79% for carbohydrates, and 17.68% for protein, with an average PMAE of 17.06%. Compared to the worst-performing method, GoogLeNet, our method improved by 19.86%, and compared to the best method, VGG16, it improved by 1.14%. Specifically, PMAE for calories improved by 0.53–12.19%, for mass by 0.12–12.52%, for fat by 2.77–23.24%, and for carbohydrates by 1.56–22.6%.

In the overall network architecture, using UNet for food image segmentation effectively eliminated the interference of background information in predicting food components. ResNet101 was able to capture rich feature representations, effectively avoiding gradient vanishing and accelerating network convergence. The SE module emphasized important features in the channels and suppressed less useful features. Finally, fully connected layers were used to predict the five components: mass, calories, fat, carbohydrates, and protein. This overall architecture not only improved prediction accuracy but also demonstrated good generalization ability.

**FIGURE 8:**
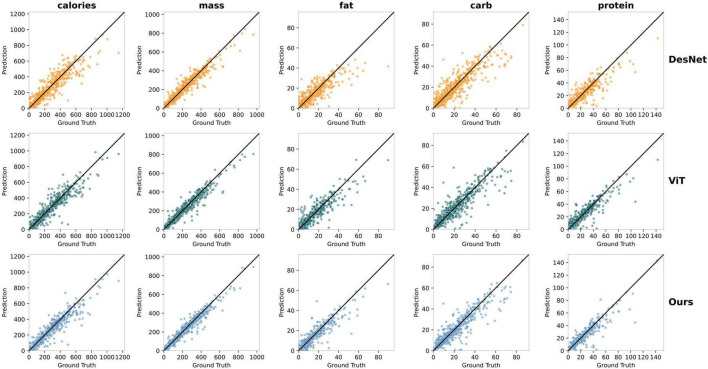
Comparison of the predicted values and actual values for five components between our method, ViT, and DenseNet.

Next, we compared the model’s predicted values with the actual values and plotted scatter plots, as shown in Figure 8. In addition to our final model, we also compared the results of the Transformer-based ViT16 and the convolutional neural network DenseNet. Each row in Figure 8 represents the prediction results of a model, with DenseNet, Vision Transformer base 16, and our method from top to bottom. Each column represents a food component, showing the performance of the three different models in predicting food calories, mass, fat, carbohydrates, and protein from left to right. In each sub-figure, the *x*-axis represents the actual values, and the *y*-axis represents the predicted values, with the black diagonal line indicating the ideal prediction line (i.e., predicted values equal to actual values). The closer the scatter points are to the black diagonal line, the closer the predicted values are to the actual values. The more concentrated and dense the scatter distribution near the diagonal line, the higher the model’s stability and accuracy, and the lower the PMAE value. Our model’s scatter distribution was more concentrated across all components, consistent with the experimental results.

Next, we analyzed the results of the models on all test samples, calculating the mean squared error (MSE), root mean squared error (RMSE), mean absolute error (MAE), and *R*-squared (*R*^2^). As shown in Table 3, the errors for calories and mass are significantly higher than those for fat, carbohydrates, and protein due to their larger magnitudes. However, by using the PMAE metric to calculate the errors, we balanced the differences caused by varying magnitudes, allowing a more intuitive view of the actual accuracy of each component’s prediction.

**TABLE 3:** Evaluation results of our method using mean squared error (MSE), root mean squared error (RMSE), mean absolute error (MAE), and *R*-squared (*R*^2^ ).

	MSE	RMSE	MAE	_*R^2*_
Calories	3,875.84	62.26	37.03	0.9
Mass	1,306.14	36.14	23.3	0.94
Fat	20.58	4.54	2.39	0.87
Carb	33.29	5.77	3.42	0.86
Protein	39.19	6.26	2.9	0.89

Additionally, we selected several comparison models to evaluate our model in terms of parameter count (Params) and computational cost (GFLOPs), as shown in Table 4. Our model has 51.96 M parameters, falling between ResNet50 (33.62 M) and ViTb16 (109.23 M). Although the parameter count is slightly higher than that of the lightweight MobileNetv3 (23.72 M), our model achieves a significant improvement in accuracy. Meanwhile, compared to models with similar accuracy, such as VGG (149.38 M) and ViTb16 (109.23 M), our model substantially reduces the parameter count, demonstrating superior efficiency. In terms of GFLOPs, our model achieves 10.28 GFLOPs, which is also at a moderate level, slightly higher than ResNet50 (9.07 GFLOPs) but far lower than VGG (20.35 GFLOPs) and ViTb16 (16.87 GFLOPs). Considering the three metrics—Mean PMAE, parameter count, and GFLOPs—our method achieves a better balance between performance and computational resource consumption, showcasing strong overall competitiveness.

**TABLE 4:** Comparison of Mean PMAE, GFLOPs, and Params between our method and comparison models.

	Mean PMAE (%)	GFLOPs	Params (M)
VGG	18.52	20.35	149.38
ViTb16	19.52	16.87	109.23
resnet50	18.33	9.07	33.62
DenseNet	23.14	3.85	29.75
MobileNetv3	21.62	3.80	23.72
Ours	17.06	10.28	51.96

**FIGURE 9:**
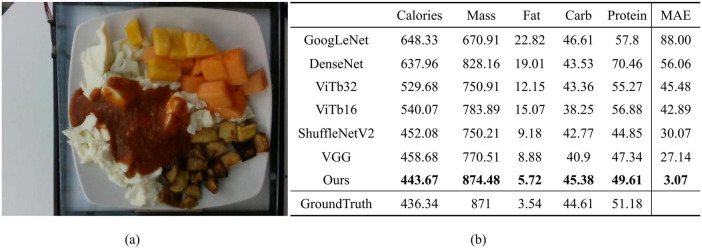
**(a)** The food image corresponding to dish_1563984296; **(b)** The specific results of nutritional component predictions for this food image using the proposed method and comparison methods, including the mean absolute error (MAE) between the predicted and ground truth values for five nutritional components. Bolded values in the table indicate the predictions closest to the ground truth.

To better demonstrate the effectiveness of our proposed method in practical nutrition prediction, we compared the actual prediction results of a specific food image, dish_1563984296, across all comparison methods, as shown in Figure 9. Figure 9a displays the food image used for prediction, which contains multiple ingredients, and Figure 9b presents a table detailing the prediction results for various nutritional components, including calories, mass, fat, carbohydrates, protein, and the mean absolute error (MAE) compared to the ground truth. The last row of the table contains the ground truth values as a reference. It can be observed that our model achieves an MAE of 3.07, significantly lower than other methods. For instance, ShuffleNetV2 and VGG, which have relatively high accuracy, report MAEs of 30.07 and 27.14, respectively, while DenseNet and ViTb16 reach MAEs as high as 60.24 and 42.89. This highlights the superior overall prediction accuracy of our model. For each specific nutritional component, the predictions from our method are also the closest to the ground truth. These results indicate that our approach can more accurately predict the nutritional components of food and demonstrates strong practical value, particularly when applied to real-world food images.

In experiments that also used top-view images from the Nutrition5k dataset for nutrient prediction, the method by Shao et al. (18) is highly representative. They used both the original RGB images and depth images, integrating multimodal feature fusion (MMFF) and multi-scale fusion for vision-based nutrition assessment. Since both their method and ours are based on Nutrition5k and use the same evaluation metric, PMAE, we conducted a comprehensive comparison between the two approaches, as shown in Table 5.

**TABLE 5:** Comparison of our method with models using RGB-D fusion for prediction.

Methods	Calorie PMAE (%)	Mass PMAE (%)	Fat PMAE (%)	Carb PMAE (%)	Protein PMAE (%)	Mean PMAE (%)
RGB-D fusion	**15.0**	**10.8**	23.5	22.4	21.0	18.5
Ours	15.68	11.75	**21.41**	**18.79**	**17.68**	**17.06**

The bold values represent the best value across all methods.

The reason for comparing our method with this approach is that both use the same dataset and evaluation metrics, making the comparison more realistic. However, the results of this depth image fusion method are heavily influenced by the quality of the depth images. If the depth images are of poor quality or inaccurate, it may adversely affect the subsequent feature fusion process, thereby impacting the accuracy and stability of nutrient prediction. In contrast, our method does not rely on depth images and depends solely on food images. As a result, it can maintain stable performance even in scenarios where depth information is missing or inaccurate.

From the experimental results, our method outperforms the depth-image fusion method in average PMAE. For specific metrics, our method significantly surpasses the RGB-D fusion method in predicting carbohydrates and protein. The segmentation-then-prediction method helps capture fine-grained features within the food, whereas the RGB-D fusion method focuses more on depth information, which can be influenced by background and other irrelevant information, potentially overlooking some details in the original image. For calorie and fat prediction, the differences between the two methods are minimal. However, in mass prediction, our method is less accurate than the RGB-D fusion method. The planar image method, compared to the depth information fusion method, might miss some overall shape or structural features, leading to lower accuracy in mass prediction.

Additionally, the depth image fusion method from Shao et al. (18) performs multi-level feature extraction and fusion for the original image and depth map, resulting in higher computational overhead and making it less suitable for resource-limited environments or real-time applications. In contrast, our method features a more streamlined structure, requiring only a single backbone network for image feature processing, followed by an SE module for attention-based feature weighting, thus reducing unnecessary computational redundancy. To demonstrate the differences in model complexity, we compared the parameters and GFLOPs metrics of our method with the RGB-D fusion model in Table 6. The results show that our model significantly outperforms the RGB-D fusion model. In terms of parameter count, our model has approximately half the parameters of the RGB-D fusion model, and in terms of floating-point operations, our model’s GFLOPs are only about 15% of the RGB-D fusion model. This indicates that our model requires less memory during training and inference, operates faster, and also achieves higher average performance metrics.

**TABLE 6:** Comparison of our method with the RGB-D fusion method in terms of Params and GFLOPs.

Method	Params (M)	GFLOPs
RGB-D fusion	110.87	68.96
Ours	**51.96**	**10.28**

The bold values represent the best value across all methods.

Additionally, the method combining color and depth images requires specialized equipment to collect depth data, which is difficult for the average person to obtain. Thus, predicting directly using color images is more feasible.

#### 3.2.3 Ablation experiment

To rigorously validate the effectiveness of our model design, we conducted a series of ablation experiments by comparing the performance of models using original RGB images versus segmented images, as well as evaluating the impact of incorporating the SE (Squeeze-and-Excitation) module. The experiments were aimed at investigating how each component influences the model’s performance in predicting nutrition values, including calories, mass, fat, carbohydrates, and protein.

We measured the PMAE (percentage mean absolute error) for each nutrient, along with the average PMAE across all nutrients. The results of the experiments are summarized in Table 7. Four different experimental combinations were tested, varying the image input type (Original vs. segmented) and the use of the SE module (with or without).

**TABLE 7:** Results of the ablation study.

Dataset	SE	Calorie PMAE (%)	Mass PMAE (%)	Fat PMAE (%)	Carb PMAE (%)	Protein PMAE (%)	Mean PMAE (%)
Original	√	18.52	15.09	25.84	23.49	24.77	21.54
	×	16.34	12.85	25.10	20.51	24.16	19.79
Segmented	√	16.08	12.08	22.73	18.66	17.98	17.51
	×	**15.68**	**11.75**	**21.41**	**18.79**	**17.68**	**17.06**

Comparison of the impact of using original images or segmented images and whether or not using the SE module on the experimental results. The bold values represent the best value across all methods.

When the SE module was kept consistent, models using segmented images as input showed improved performance over those using the original RGB images. The improvement in average PMAE ranged from 2.73 to 4.03%. This suggests that segmenting the food items helped to reduce the influence of background noise and irrelevant elements, thereby improving the precision of nutrition prediction. Segmenting the food regions appears to help the model focus on relevant information, which contributes to the performance gains observed.

When the input dataset was fixed, incorporating the SE module resulted in an improvement in average PMAE, ranging from 0.45 to 1.75%. This result indicates that the SE module enhances the model’s ability to emphasize critical features in the channel dimensions, leading to more accurate nutrient estimation. The SE module likely improves feature extraction by weighting more important channels, which has a positive effect on prediction accuracy.

## 4 Discussion

We based our study on the Nutrition5k dataset, selecting 3,224 usable images from the original 3,490 top-down images. We manually annotated the food portions with segmentation labels and saved various segmentation results, including JSON files, foreground images, binary masks, and pseudo-color images. These segmented images, along with their detailed nutrition labels, were used for the nutrition prediction task.

First, we trained the UNet model for segmentation using the labeled data. The model achieved Dice, Jaccard, Precision, and Recall scores of 97.69, 95.52, 97.40, and 98.02%, respectively, indicating that the segmented results were very close to the ground truth.

Next, we used the segmented pure food images to train the nutrition prediction model. We compared various backbones and found that the model with ResNet101 as the backbone achieved the best performance. The PMAE for calories, mass, fat, carbohydrates, and protein were 15.68, 11.75, 21.41, 18.79, and 17.68%, respectively, with an average PMAE of 17.06%. This represents an improvement of 1.14 to 19.86% over other comparison models.

The predictions of calorie, mass, carbohydrate, protein, and fat content from our model can help in scientifically planning diets. Here are some guidelines:

1. Calculate the Basal Metabolic Rate (BMR) and Total Daily Energy Expenditure (TDEE) based on age, gender, weight, height, and activity level.
2. Set daily caloric and macronutrient (protein, fat, carbohydrate) intake goals based on health objectives such as weight loss, muscle gain, or weight maintenance.
3. After preparing food, use photo-based prediction to calculate the nutritional content of the meal and determine if it meets the set goals. Adjust the food proportions or make appropriate changes for the next meal based on the results to ensure daily nutritional intake aligns with the goals.
4. Regularly record diet and weight changes to monitor progress and adjust the diet plan as needed.

Despite these positive results, our work is subject to several practical limitations that may impact its performance in real-world scenarios:

1. First, since the Nutrition5k dataset was captured under controlled conditions with uniform lighting and minimal noise, images taken in real-world settings using smartphones under varying conditions may lead to decreased model performance. To address this, we have applied data augmentation to improve the dataset’s generalizability, but a larger dataset or more robust models could further enhance performance in such cases.
2. Secondly, the dataset comes from a single restaurant with limited food types, and the model’s performance may decline when applied to food types beyond the dataset. Future work could focus on training models on more diverse datasets, capturing different cuisines, meal presentations, and environmental settings, to improve the model’s robustness and applicability across various real-world scenarios.
3. Finally, in real life, food items are often stacked or mixed together, making them harder to detect and separate. In practical applications, undetected or inaccurately segmented food items may lead to incorrect nutrition predictions. For instance, a bowl of mixed salad may be more difficult for the model to segment compared to the neatly arranged portions in Nutrition5k. Techniques such as multi-instance segmentation and hierarchical labeling could help improve segmentation accuracy in these complex food arrangements.

While our model demonstrated effective nutrition prediction in this study, additional advancements are necessary for practical, real-world dietary monitoring. For instance, integrating continuous learning methods could allow the model to improve as it is exposed to more varied data from different users. Additionally, developing user-friendly mobile applications with built-in real-time correction for segmentation or prediction errors would further enhance the practical value of this work.

## 5 Conclusion

To help ordinary people better understand and manage their diets, we propose a food image nutrition prediction scheme based on a segmentation-regression method. This study is based on the Nutrition5k dataset, where we manually annotated the food images with segmentation labels and combined them with detailed nutritional content labels to construct a segmentation-regression model for nutrition prediction.

Our model achieved an average PMAE of 17.45% across multiple nutrient predictions, validating its effectiveness and accuracy. Our method employs the UNet model for image segmentation, uses a backbone network to extract features, and enhances feature representation through the SE structure. This approach enables accurate prediction of weight, calories, fat, carbohydrates, and protein.

Our research not only addresses the gap in current methods regarding comparison with real nutritional labels but also provides reliable technical support for future vision-based food nutrition evaluation. The manually annotated segmentation labels are now publicly available for use in subsequent research.

## Data Availability

The datasets presented in this study can be found in online repositories. The names of the repository/repositories and accession number(s) can be found below: https://doi.org/10.6084/m9.figshare.26252048.v1.
